# Epidemiology and psycho-social aspects of onchocercal skin diseases in northeastern Nigeria

**DOI:** 10.1186/1475-2883-6-15

**Published:** 2007-12-03

**Authors:** Ikem Chris Okoye, Celestine OE Onwuliri

**Affiliations:** 1 Department of Zoology, University of Jos, Nigeria; 2 (current address) Applied Parasitology Unit, Department of Zoology, University of Nigeria, Nsukka, Enugu State, Nigeria; 3 (current address) Office of the Vice-Chancellor, Federal University of Technology, Owerri, Imo State, Nigeria

## Abstract

**Background:**

Observations were made on the prevalence of onchocerciasis and Onchocercal Skin Diseases (OSD); frequency of occurrence and anatomical distribution of OSD in the Hawal River Valley, an established onchocerciasis endemic focus in north-eastern Nigeria.

**Methods:**

Symptoms of OSD were diagnosed in 5 844 subjects using Rapid Assessment Method (RAM) while 1 479 of the subjects chosen from alternate households had their skin biopsies examined for active microfilariae of *Onchocerca volvulus*. Also, Focal Group Discussions (FGD) were conducted at the Health District levels.

**Results:**

*O. volvulus *was recorded in (19.0%) and OSD in (43.8%) of the subjects. The Mantel-Haenszel test for linear association showed a close agreement between onchocerciasis prevalence and the rate of OSD (χ^2 ^= 3.93; p < 0.05). The various forms of OSD occurred in the order: CPOD (17.7%), APOD (9.9%), DPM (9.0%), LOD (7.0%) and ATR (3.1%). The overall frequency of occurrence of various symptoms of OSD on different anatomical locations showed the locations in descending order of occurrence as lower limbs (24.6%), upper limbs (21.3%), buttocks (19.9%), shoulder & neck (19.1%), abdomen and trunk (11.3%), backside (10.6), and 'other' sites (7.5%). The Focal Group Discussion (FGD) revealed the most worrisome consequences of OSD as social isolation of victims (31.3%), shame and low self esteem (22.7%) and high cost of medication (15.6%).

**Conclusion:**

It is recommended that Onchocerciasis control programmes in the Hawal River Valley and any other focus with high incidence of OSD should incorporate an aspect that would address the anxiety and depression caused by various OSD lesions since they carry lots of psycho-social implications. This would increase acceptance and compliance of the target population. The classification criteria of onchocerciasis endemicity should be based on either or both of the *O. volvulus *and onchocercal skin disease burden of any community and no longer on *O. volvulus *parasitic infection rate alone.

## Background

Onchocerciasis or River blindness is a chronic multisystem disease caused by infection of a parasitic nematode – *Onchocerca volvulus*. The symptomatology of onchocerciasis has revealed the clinical manifestations as onchocerca skin diseases, onchocercomata, lymphadenopathy and ocular lesions, including the irreversible terminal effect of blindness. The disease is endemic in Africa, Latin America and Yemen [1]. It has a high damaging potential to the social life of patients especially the stigmatising premature ageing and lizard skin presentation.

Onchodermatitis is usually the first visible symptom of onchocerciasis. It usually begins with intense itching and progressing to a manifestation of irritating papular rashes known as *craw-craw *in parts of Africa. This acute papular dermatitis presents with small pruritic papules that may develop into pustules or vesicles. The condition could later deteriorate into chronic papular onchodermatitis that present large papules and may lead to hyper-pigmentation and the thickening of the skin. This is usually followed by lichenification of the skin resulting in mosaic patterns popularly known as *lizard skin *(*crocodile *skin or sowda) while the advanced stage is characterised by depigmentation known as *Leopard skin*, loss of elasticity and atrophy of the skin [2].

Onchocerciasis may not directly cause death but it carries great social and economic consequences. OSD is a leading cause of morbidity in endemic areas, resulting in psycho-social consequences and isolation. The disease burden due to onchocerciasis has been estimated at 884 000 Disability Adjusted Life Years (DALYs), 60% of which is accounted for by OSD [3]. Unfortunately, to-date, less emphasis is placed on OSD control in onchocerciasis endemic foci with blinding onchocerciasis such as the Hawal River Valley where OSD also exerts great disease burden.

This paper reports details of striking observations made on the manifestations and anatomical locations of cases of onchocerca skin diseases and the relationship between *O. volvulus *and OSD during a wider study on the epidemiology, socio-economic effects and control of onchocerciasis in parts of north eastern Nigeria.

## Methodology

### The study area

The study area is the Hawal River Valley which lies within the southern border of Borno and northern part of Adamawa states. The area has been well known for onchocerciasis endemicity and an isolated focus on the border of Borno and Adamawa provinces that is heavily infested with the *Simulium *vectors and where a heavy intensity of onchocerciasis occurs [4]. It is undoubtedly the most serious onchocerciasis endemic focus within North – Eastern Nigeria and lies along the valley of the Hawal River down-stream from Garkida. Studies in the area have recorded serious ocular and socio-economic toll of the disease [5-7].

## Subjects and method

### Enlistment of subjects and communities surveyed

A total of 5 844 subjects from 55 community clusters chosen by the Rapid Epidemiological Mapping for Onchocerciasis (REMO) by Ngoumou and Walsh, [8] were examined for OSD, a clinical symptom of Onchocerciasis by the Rapid Assessment Method (RAM). A quarter (1 479) of the subjects chosen from alternate households had their skin biopsies examined for microfilariae of *Onchocerca volvulus*. The villages were made the primary unit (statistical cluster) for the survey. In large villages, one or more wards were selected using purposive sampling as described by Hammon [9] and the entire population examined to avoid the usual displeasure of those omitted. Informed consent of the subjects was obtained through their heads of households before skin biopsies were made.

### Examination of skin biopsy

The procedures of the Onchocerciasis Control Programme (OCP) in West Africa as detailed by Akogun and Onwuliri [10] were used. Bloodless skin biopsies were taken from either sides of the iliac crest using the German-made Holth type corneosclera punch with 1.5 mm bite after cleaning the site with cotton swabs moistened with 70% ethanol. Each skin fragment was placed in polystyrene microtitration plate with U-shaped wells containing 0.3 ml physiological saline. The wells of the completely filled plates were covered with adhesive tapes to prevent evaporation and spilling of contents during transportation.

### Clinical survey

The Rapid Assessment Method (RAM) used for clinical examination was that of Ngoumou and Walsh (1993). The observed OSD were identified and classified as Acute Papular Onchodermatitis (APOD); Chronic Papular Onchodermatitis (CPOD); Lichenified Onchodermatitis (LOD); Atrophy (ATR) or Depigmentation (DPM) according to the grading system for cutaneous changes in onchocerciasis given by Murdoch et. al. [11].

### Focus group discussion (FGD)

Two FGDs were conducted in each of the health districts to fill in the gaps in knowledge and obtain more detailed information on the subject matters raised including follow-ups towards achieving the research objectives.

#### In-depth interviews

In-depth interviews were conducted to obtain more detailed information on the issues under study.

## Results

### Skin biopsy and clinical survey

The overall prevalence of Onchocerciasis was 19.0% and that of OSD was 43.8%. Males had higher prevalence of *O. volvulus *(19.6%) and OSD (44.2%) than females (18.3 % and 43.3% respectively) though the differences were not statistically significant (p > 0.05). The rate of OSD increased with age for both sexes up to 31–40 years age group and then, decreased slightly- Table 1.

**Table 1:** Relationship between the Prevalence of Onchocerca volvulus microfilariae and Onchocerca Skin Disease (OSD) among the Residents of Hawal River Valley

	**Males**			**Females**			**Both Sexes**		
**Age Group**	**No. Exam**	**No. mf +ve**	**No. OSD +ve**	**No. Exam**	**No. mf +ve**	**No. OSD +ve**	**No. Exam**	**No. mf +ve**	**No. OSD +ve**

**(Years)**	**(No. SS)**	**(%)**	**(%)**	**(No. SS)**	**(%)**	**(%)**	**(No. SS)**	**(%)**	**(%)**

**≤ 10**	461(114)	18(15.8)	169(36.7)	417(89)	13(14.6)	153(14.6)	878(203)	31(15.3)	322(36.7)
**11 20**	447(108)	19(17.6)	184(41.2)	454(111)	20(18.0)	181(39.9)	901(219)	39(17.8)	365(40.5)
**21 – 30**	523(127)	26(20.5)	220(42.1)	461108)	19(19.6)	198(41.0)	984(235)	45(19.1)	409(41.6)
**31 – 40**	556(142)	30(21.1)	285(51.3)	450(124)	26(19.8)	231(51.3)	1006(266)	56(21.1)	516(51.3)
**41 – 50**	548(150)	31(20.7)	265(48.4)	498(131)	26(19.8)	230(46.2)	1046(281)	57(20.3)	495(47.3)
**≥ 51**	507(137)	28(20.7)	223(44.0)	522(140)	25(17.9)	228(43.7)	1029(275)	53(19.3)	451(43.8)

**Total**	**1042(776)**	**152(19.6)**	**1346(44.2)**	**2802(703)**	**129(18.3)**	**1212(43.3)**	**5844(1479)**	**281(19.0)**	**2558(43.8)**
	**No. SS = No. Skin Snipped**			**mf+ve = Microfilari positive**					

The Mantel-Haenszel test for linear association showed a close agreement between onchocerciasis prevalence and the rate of OSD (χ^2 ^= 3.93; p < 0.05).

As shown in Table 2, the form of OSD with the highest overall percentage frequency of occurrence was CPOD (17.7%), followed by APOD (9.9%) while ATR (3.1%) was the least. Age-dependent analysis of these symptoms showed that the rates of CPOD and LOD increased with age for both sexes, APOD decreased with age for both sexes while ATR increased with age for males but did not follow a definite pattern for females.

**Table 2:** Age and Sex related frequency of occurrence of various forms of onchocerca skin diseases among residents of Hawal river valley

	**Age Group(Years)**	**No Examined**	*Forms	**APOD**	**CPOD**	**LOD**	**ATR**	**DPM**	**Total**	
**Males**	**≤ 10**	**461**		81(17.6)	56(12.1)	18(3.9)	0(0.0)	21(4.6)	**176(38.2)**	
	**11 – 20**	**447**		68(15.2)	64(14.3)	25(5.6)	12(2.7)	25(5.6)	**194(43.4)**	
	**21 – 30**	**523**		56(10.7)	94(18.0)	31(5.9)	18(3.4)	49(9.4)	**248(47.4)**	**n = 1346**
	**31 – 40**	**556**		44(7.9)	101(18.2)	49(8.8)	20(3.6)	54(9.7)	**268(48.2)**	
	**41 – 50**	**548**		31(5.7)	108(19.7)	50(9.1)	23(4.2)	55(10.0)	**267(48.1)**	
	**≥ 50**	**507**		20(3.9)	88(17.4)	50(9.9)	23(4.5)	54(10.7)	**235(46.4)**	

	**Total**	**3042**		**300(9.9)**	**511(16.8)**	**223(1.3)**	**96(3.2)**	**258(8.5)**	**388(45.6)**	

**Females**	**≥ 10**	**417**		79(18.9)	54(12.9)	15(3.6)	0(0.0)	30(7.2)	**178(42.7)**	
	**11 – 20**	**454**		67(14.8)	71(15.6)	23(5.1)	10(2.2)	38(8.4)	**209(46.0)**	
	**21 – 30**	**461**		46(10.0)	87(18.9)	24(5.2)	20(4.3)	45(9.8)	**22(48.2)**	**n = 1212**
	**31 – 40**	**450**		42(9.3)	108(24.0)	28(6.2)	17(3.8)	47(10.4)	**42(53.2)**	
	**41 – 50**	**498**		25(5.0)	104(20.9)	47(9.4)	19(3.8)	52(10.4)	**47(49.6)**	
	**≥ 50**	**522**		17(3.3)	99(19.0)	50(9.6)	22(4.2)	55(10.5)	**43(46.6)**	

	**Total**	**2802**		**276(9.9)**	**523(18.7)**	**187(6.7)**	**88(3.1)**	**267(9.5)**	**341(47.9)**	

• Subjects with two or more forms encountered.

APOD = Acute Papular Onchocerca Dermatitis.

CPOD = Chronic Papular Onchocerca Dermatitis.

LOD = Lichenified Onchocerca Dermatitis.

ATR = Atrophy.

DPM = Depigmentation

The anatomical distribution of the various OSD decreased from 24.6% on the lower limbs to 10.0 % on the backside (Table 3).

**Table 3:** Sex related prevalence of onchocerca skin diseases on different anatomical locations of residents of the Hawal river valley

	**Forms**	*Location	**Shoulder & Neck**	**Buttocks**	**Lower Limbs**	**Upper Limbs**	**Abdomen &Trunk**	**Backside**	**Others	
	**APOD**		53	42	115	125	26	34	21	
	**CPOD**		87	99	50	36	60	50	39	
	**LOD**		34	32	60	51	26	21	17	
**Males**	**ATR**		22	33	31	26	23	14	0	**n = 1346**
	**DPM**		61	61	70	55	23	24	20	

	**Total(%)**		**257(19.1)**	**267(19.8)**	**326(24.2)**	**294(21.8)**	**158(11.7)**	**143(10.6)**	**97(7.2)**	

	**APOD**		36	34	132	116	23	30	25	
	**CPOD**		99	98	53	44	50	51	37	
	**LOD**		28	27	42	30	19	17	14	
**Females**	**ATR**		16	29	13	15	17	12	0	
	**DPM**		52	54	63	46	22	19	20	**n = 1212**

	**Total(%)**		**231(19.1)**	**242(20.0)**	**303(25.0)**	**249(20.5)**	**131(10.8)**	**129(10.6)**	**96(7.9)**	

**Both sexes**	**APOD**		89	76	247	241	49	64	46	
	**CPOD**		186	197	103	80	110	101	76	
	**LOD**		62	59	102	81	45	38	31	
	**ATR**		38	62	44	41	40	26	0	
	**DPM**		114	116	133	101	45	43	40	

	**Total(%)**		**489(19.1)**	**510(19.9)**	**629(24.6)**	**544(21.3)**	**289(11.3)**	**272(10.6)**	**193(7.5)**	

*Multiple location of single cases encountered.

**Head, face and chest.

APOD = Acute Papular Onchocerca Dermatitis.

CPOD = Chronic Papular Onchocerca Dermatitis.

LOD = Lichenified Onchocerca Dermatitis.

ATR = Atrophy.

DPM = Depigmentation

Focal group discussions & In-depth interviews

The discussants were asked to state what they perceived as the most worrisome consequence of OSD. The responses are shown in Table 4. Social isolation topped the list (31.3%), followed by shame and low self-esteem (22.7%) while 'others' viz. musculo-skeletal pains, fever and divorce was least (3.9%).

**Table 4:** Most Worrisome Consequence of OSD in Hawal river valley, Nigeria (n = 128)

**Effect**	**No. (%) Responses**
Social isolation (Hindrance to social interaction)	40(31.3)
Shame and low self esteem ('self-hatred')	29(22.7)
High cost of medication	20(15.6)
Skin blemishes	15(11.7)
Restlessness/Sleeplessness	11(8.6)
Marital problems	8(6.3)
Others (Pains, headache, fever)	5(3.9)

The In-depth interviews yielded some qualitative results as depicted by the quotes below:

*I am always afraid (anxious) that an attack of (intermittent) itching in a private part (buttocks, waist, groin) could occur at a public gathering; I therefore kept off; infact, I hated myself"*. – 48 year-old, once affected, female participant.

*"When a lady's body has been spoilt by 'mbiba' (papular rashes), only elderly widowers and already married men would seek her hand in marriage"*. Affected, 26 year-old, FGD participant

*"I was unable to either walk or bend during the last weeding period because of itching, body pains and fever"*. – 53 year-old, male farmer.

## Discussion

The prevalence of *onchocercaa volvulus *correlated closely with the severity of OSD (χ^2 ^= 3.93; p < 0.05) by the Mantel-Haenszel test for linear association. The implication is that OSD or its most frequent form, CPOD, could be a clinical diagnostic index for estimating *O volvulus *endemicity.

The decrease of the rate of APOD with age could be due to the fact that it is only common in early infections, being intermittent and disappearing in long standing heavy infections in which many microfilariae may have died [12]. The opposite inference could be drawn for the increase of APOD with age (Table 3). It could also be that in older age groups, the parasite and the host's body have reached a state of equilibrium, which may reduce cases of acute irritations to low levels. The reason of super-infection would explain the progressive deterioration of APOD and CPOD to LOD, DPM and ATR. It would also account for the increase of the severity of these three symptoms with increase in age.

The preferred sites for the various OSD manifestations reveals some reasons for the psycho-social inclinations of the victims. The limbs which are usually largely exposed and the areas of the body considered 'private' viz. buttocks & groin were heavily infested with various OSD symptoms. Lesions of acute and chronic papular dermatitis (APOD and CPOD) are embarrassing features when visible on the exposed parts of the bodies of adolescent boys and girls. The lichenification caused by LOD on the limbs, shoulder and neck of victims and the depigmentation (leopard skin) on the shin, shoulder and neck are cosmetic blemishes. These lesions are usually repulsive and often hindrances to free social interaction by victims. The above could account for the position of the majority of FGDs who were worried about the social isolation suffered by people affected by OSD.

## Conclusion

It is recommended that Onchocerciasis control programmes in the Hawal River Valley and any other focus with high incidence of OSD should incorporate an aspect that would address the anxiety and depression caused by various OSD lesions since they carry lots of psycho-social implications. This would increase acceptance and compliance of the target population.

The classification criteria of onchocerciasis endemicity in parts of Africa should be based on either or both of the *O. volvulus *and onchocercal skin disease burden of that community and no longer on *O. volvulus *parasitic infection alone. Already in Yemen, the prevalence of *sowda *serves as an alternative marker to nodule measurement [13]. Nodule rate is the endemicity marker for onchocerciasis in the Rapid Epidemiological Assessment (REA) method.

## Authors' contributions

ICO designed the study, carried out the skin biopsy with the assistance of Laboratory Technologists and performed the statistical analysis. COE conceived and supervised the study. ICO and CEO carried out the clinical examinations, Focus group discussions and the in-depth interviews. Both authors read and approved the final manuscript.
